# Supplementary material for: Comparative genomics provides new insights into the diversity, physiology, and sexuality of the only industrially exploited tremellomycete: Phaffia rhodozyma
Source: BMC Genomics. 2016 Nov 9;17:901. doi: 10.1186/s12864-016-3244-7 (PMC5103461; doi:10.1186/s12864-016-3244-7)
Supplement: Additional file 6: — List of orphan genes with links to PFAM (related to Additional file 1: Table S1). (ZIP 1428 kb) [file 12864_2016_3244_MOESM6_ESM.zip › PFAM_UNIQUE/G01663_P.html]

#                                                                            --- full sequence --- -------------- this domain -------------   hmm coord   ali coord   env coord
  
# target name        accession   tlen query name           accession   qlen   E-value  score  bias   #  of  c-Evalue  i-Evalue  score  bias  from    to  from    to  from    to  acc description of target
  
#------------------- ---------- ----- -------------------- ---------- ----- --------- ------ ----- --- --- --------- --------- ------ ----- ----- ----- ----- ----- ----- ----- ---- ---------------------
  
G01663\_P             -            425 RunxI                PF08504.6    101       7.6    5.7   7.1   1   3    0.0001      0.61    9.2   0.3    37    96   144   202   130   205 0.72 - Runx inhibition domain  
G01663\_P             -            425 RunxI                PF08504.6    101       7.6    5.7   7.1   2   3      0.51     3e+03   -2.6   1.5    76    76   331   331   288   392 0.61 - Runx inhibition domain  
G01663\_P             -            425 RunxI                PF08504.6    101       7.6    5.7   7.1   3   3      0.31   1.9e+03   -1.9   0.2    50    50   380   380   333   418 0.53 - Runx inhibition domain
